# How different of the rhizospheric and endophytic microbial compositions in watermelons with different fruit shapes

**DOI:** 10.1371/journal.pone.0302462

**Published:** 2024-05-16

**Authors:** Jian Xiao, Jinyan Huang, Kezhuo Xiao, Guifen Li, Shangdong Yang, Yi He

**Affiliations:** 1 Guangxi Key Laboratory of Agro-Environment and Agro-Products Safety, National Demonstration Center for Experimental Plant Science Education, Agricultural College, Guangxi University, Nanning, 530004, P. R. China; 2 Longping Branch, College of Biology, Hunan University, Changsha, 410125, Hunan, P. R. China; 3 Horticultural Research Institute, Guangxi Academy of Agricultural Sciences, Nanning, 530007, Guangxi, P. R. China; Osmania University, INDIA

## Abstract

Fruit shape is an important character of watermelon. And the compositions of rhizospheric and endophytic microorganisms of watermelon with different fruit shape also remains unclear. To elucidate the biological mechanism of watermelon fruit shape formations, the rhizospheric and endophytic microbial community compositions between oval (OW) and circular watermelons (CW) were analyzed. The results showed that except of the rhizospheric bacterial richness (*P* < 0.05), the rhizospheric and endophytic microbial (bacterial and fungal) diversity were not statistically significant between OW and CW (*P* > 0.05). However, the endophytic microbial (bacterial and fungal) compositions were significantly different. Firstly, *Bacillus*, *Rhodanobacter*, *Cupriavidus*, *Luteimonas*, and *Devosia* were the unique soil dominant bacterial genera in rhizospheres of circular watermelon (CW); In contrast, *Nocardioides*, *Ensifer*, and *Saccharomonospora* were the special soil dominant bacterial genera in rhizospheres of oval watermelons (OW); Meanwhile, *Cephalotrichum*, *Neocosmospora*, *Phialosimplex*, and *Papulaspora* were the unique soil dominant fungal genera in rhizospheres of circular watermelon (CW); By contrast, *Acremonium*, *Cladosporium*, *Cryptococcus_f__Tremellaceae*, *Sodiomyces*, *Microascus*, *Conocybe*, *Sporidiobolus*, and *Acremonium* were the unique soil dominant fungal genera in rhizospheres of oval watermelons (OW). Additionally, *Lechevalieria*, *Pseudorhodoferax*, *Pseudomonas*, *Massili*a, *Flavobacterium*, *Aeromicrobium*, *Stenotrophomonas*, *Pseudonocardia*, *Novosphingobium*, *Melittangium*, and *Herpetosiphon* were the unique dominant endophytic bacterial genera in stems of CW; In contrast, *Falsirhodobacter*, *Kocuria*, and *Kineosporia* were the special dominant endophytic genera in stems of OW; Moreover, *Lectera* and *Fusarium* were the unique dominant endophytic fungal genera in stems of CW; By contrast, *Cercospora* only was the special dominant endophytic fungal genus in stems of OW. All above results suggested that watermelons with different fruit shapes exactly recruited various microorganisms in rhizospheres and stems. Meanwhile, the enrichments of the different rhizosphric and endophytic microorganisms could be speculated in relating to watermelon fruit shapes formation.

## Introduction

Watermelon (*Citrullus lanatus* L.), a horticultural crop which belongs to the Cucurbitaceae family [1], is the third largest fruit crop in the world [2], and its global production is over 100 million tons [2]. China is the largest watermelon producer in the world, accounting for nearly 68% of the world’s total production [3].

Fruit shape is an important character of melon and horticultural crops, as well as an important index of product classification, grading and evaluation [4]. In addition, watermelon fruit shape has an important influence on consumer preference, packaging and transport logistics, and is also an important trait for watermelon breeding [3]. The watermelon fruit shapes were divided into round or not round (elongate and oval) [4,5]. Legendre et al. [3] found consumers typically prefer one shape of watermelon over others at the point of sale, with the current trend shifting from large, slender watermelons to smaller, blocky or round ones. Previous studies had considered that watermelon fruit shape was controlled by an incompletely dominant gene, resulting in elongated (*OO*), oval (*Oo*) and spherical (*oo*) fruits [5–7].

Recent researches have shown that microbes may emit substances that are comparable to plant hormones, such as auxins, cytokinins, abscisic acid, and gibberellins. And these substances can influence plant gene expression and thus result in physiological changes in surrounding plants [8–10]. For examples, microbes can alter the expression of genes involved in ROS scavenging and ethylene production, boosting plant growth and photosynthetic performance and allowing plants to better withstand environmental stresses, such as salinity, drought and heavy metals [8,11–13]. Also, plant gene expression can be changed by soil microbes to optimize plant responses to salt stress [8].

However, previous studies focused on screening and identifying key genes and metabolites involved in regulating watermelon fruit shape [3,14,15]. In our previous research, we explored rhizospheric and endophytic microorganisms of watermelon with different rind colors [1]. But whether rhizospheric and endophytic microorganisms are also involved in watermelon fruit shape formation, it still remains unclear. In this study, to investigate the relationship between the watermelon fruit shapes formation and their microbiomes, the rhizospheric and endophytic microbial compositions between oval and circular watermelons were analyzed.

## Materials and methods

### Experimental location, design and implementations

The field experiment was located in Suxu town, Nanning city, Guangxi Zhuang Autonomous Region, China (108° 6′ 11′′ E, 22° 28′ 28′′ N). The climate belongs to subtropical monsoon and the annual average temperature and precipitation are 21.40°C and 1213.00 mm, respectively. And the soil physicochemical properties in experimental sites are as follow: pH, 4.45; soil organic matter, 9.96 g kg^-1^; total nitrogen, 0.74 g kg^-1^; available phosphorus, 17.0 mg kg^-1^; available potassium, 78.0 mg kg^-1^.

**Fig 1:**
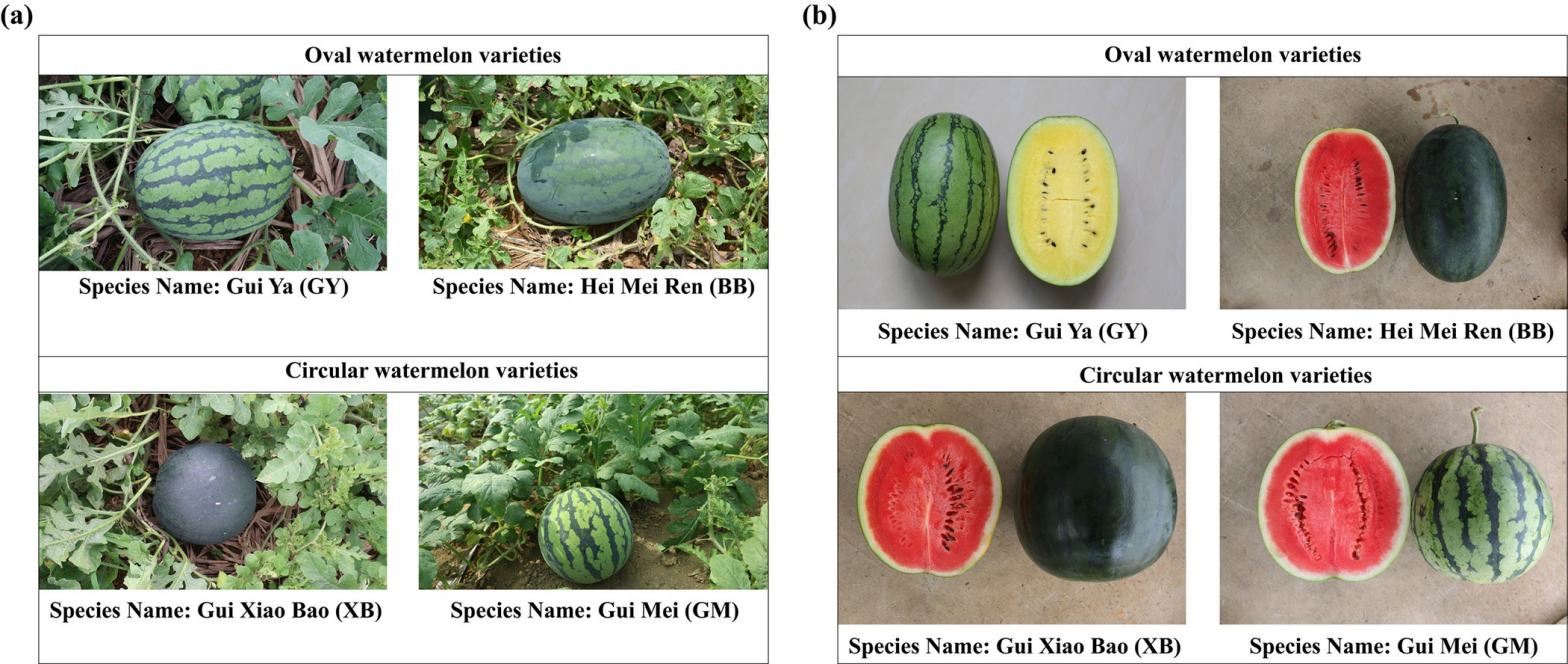
Different watermelon varieties with oval and circular shapes. **(a)** Matured watermelon fruits growing in the same field with identical management. **(b)** Cross-sectional view of the watermelon fruits.

The oval watermelon (OW) varieties, Gui Ya (GY), Hei Mei Ren (BB) and the circular watermelon (CW) varieties, Gui Mi Bao (XB), Gui Mei (GM) were used for analysis from Horticultural Research Institute, Guangxi Academy of Agricultural Sciences **(Fig 1)**. All watermelon varieties were randomly planted in the same time and grew in the same filed at February, 2021 under identical managements. Each plot area was 15.0 m^2^, and 30 plants of every variety with 0.5 m spacing were planted in each plot.

### Soil and plant samplings

Soil and plant samples were collected at May 20, 2021.The general methods of collecting for rhizospheric and endophytic samples had been described previously in detail [1]. Background soils (BG) with no watermelon growing in them in watermelon-growing fields were collected randomly for the soil background values. The detailed sample handling process is as follows: After shaking and removing bulk soils with a sterile brush, soil attaching on the stems were collected in sterile bags and stored in an ice box, and then transported to the laboratory immediately using as the rhizosphere soils [13,16]. Also, our earlier methods were used to remove the surface impurities and adherents of the stems of watermelons, wash, and sterilize thoroughly [17,18].

### Analysis of rhizospheric and endophytic microbial diversity

The total DNA extracted, PCR amplification and sequence determination were done sequentially following previous protocols [1]. The primer names and sequences of rhizospheric and endophytic bacteria and fungi were shown in **Table 1**. The processing and analysis of sequencing data have been described previously in detail [1,17–20]. We used the NCBI Sequence Read Archive (SRA) database (Accession Number: PRJNA926101) to store the raw reads.

**Table 1:** The primer sequence of rhizospheric and endophytic bacteria and fungi.

Sequencing type	Primer name	Sequence	Regions	Amplification rounds
Rhizospheric bacterial	338F	5’-ACTCCTACGGGAGGCAGCAG-3’	V3-V4	—
	806R	5’-GGACTACHVGGGTWTCTAAT-3’		
Endophytic bacterial	799F	5’-AACMGGATTAGATACCCKG-3’	V5-V7	First round
	1392R	5’-ACGGGCGGTGTGTRC-3’		
	799F	5’-AACMGGATTAGATACCCKG-3’		Second round
	1193R	5’-ACGTCATCCCCACCTTCC-3’		
Rhizospheric and endophytic fungal	ITS1F	5’-CTTGGTCATTTAGAGGAAGTAA-3’	ITS1	—
	ITS2R	5’-GCTGCGTTCTTCATCGATGC-3’		

### Statistical analyses

Alpha diversity, Principal co-ordinates analysis (PCoA) (Bray-Curtis distance), Partial Least Squares Discriminant Analysis (PLS-DA), microbial community composition, Venn diagram analysis and Linear discriminant analysis (LDA) and an LDA effect size (LEfSe) analysis of microbial community were carried out according to our previous methods. BugBase was used for phenotypic prediction, then a two-tailed Wilcoxon rank-sum test was performed on the data, a multiple test correction for *P*-values was performed using FDR, and bootstrap was used to calculate confidence intervals. Wilcoxon rank-sum test was used to analyze the significant differences (*P* < 0.05). The Majorbio Cloud Platform (www.majorbio.com) was used to conduct online data analysis [21].

## Results

### Soil bacterial compositions in rhizospheres of watermelons with different fruit shapes

**Fig 2:**
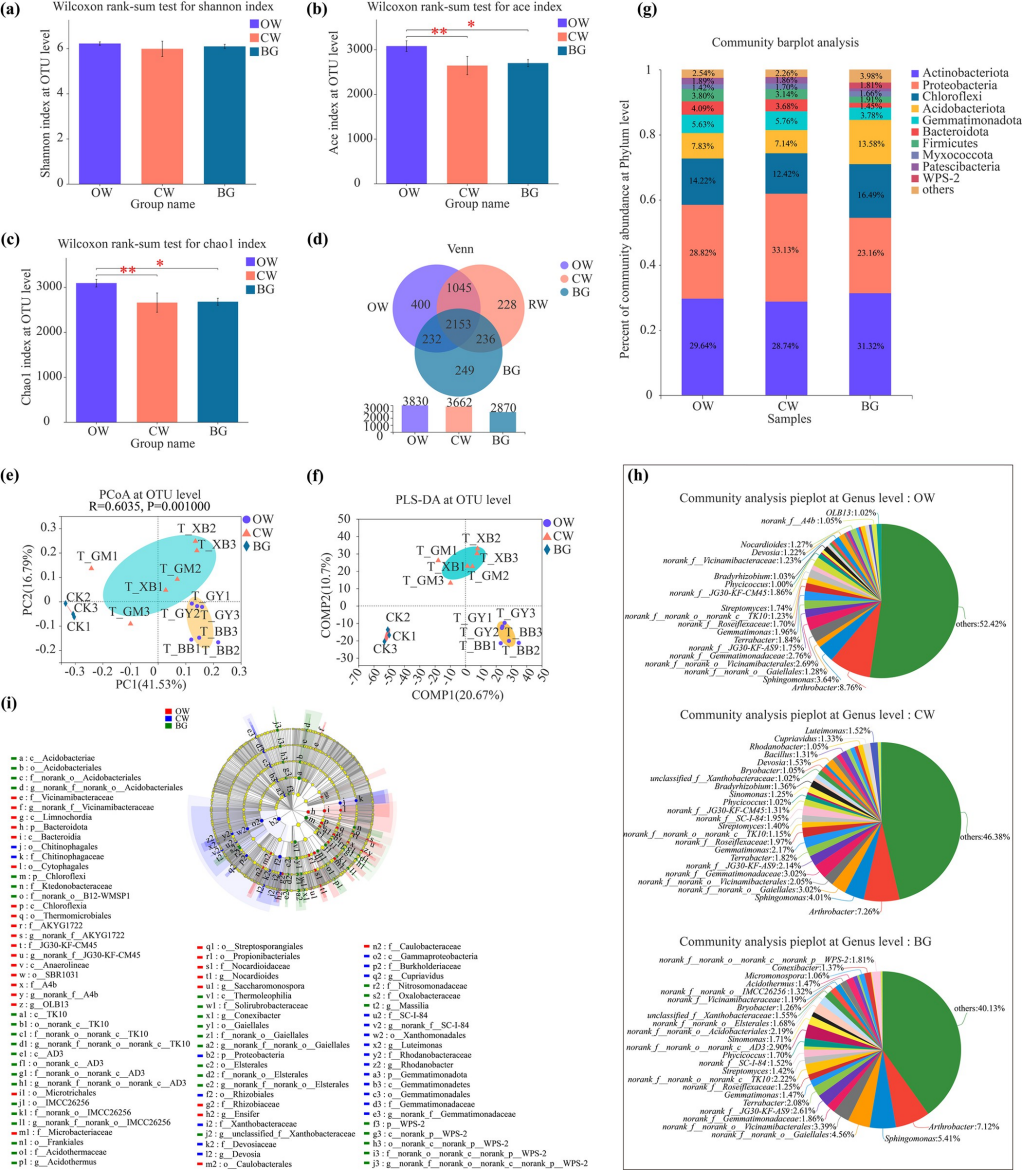
Comparison of soil bacterial community structures in rhizospheres of OW, CW and BG. **(a)** The Shannon index of soil bacterial diversity at the OTU level. **(b)** The Ace index of soil bacterial richness at the OTU level. **(c)** The Chao 1 index of soil bacterial richness at the OTU level. **(d)** PCoA of soil bacterial communities at the OTU level. **(e)** PLS-DA of soil bacterial communities at the OTU level. **(f)** Venn analyses of soil bacteria for the three treatments at the OTU level. **(g)** Compositions of dominant soil bacterial community at the phylum level. **(h)** Compositions of dominant soil bacterial community at the genus level. **(i)** LEfSe analysis of dominant soil bacteria (LDA > 3.5). *0.01 < *P* ≤ 0.05, **0.001 < *P* ≤ 0.01 using the Wilcoxon rank-sum test. The evolutionary level, from phylum to genus, is shown by circles. Each circle’s diameter varies according on how numerous the group is. Distinct prefixes (p: Phylum; c: Class; o: Order; f: Family; g: Genus) denote distinct levels. OW, oval watermelon; CW, circular watermelon; BG, background. The same as below.

The rhizospheric soil bacterial diversity (Shannon) indices were not statistically significant among OW, CW and BG (*P* > 0.05) **(Fig 2A)**. However, the Ace **(Fig 2B)** and Chao1 **(Fig 2C)** indices of rhizospheric soil bacterial richness were extremely significantly or significantly higher in rhizospheres of OW than those of CW and BG (*P* < 0.05). In addition, the results of Venn analyses showed that the numbers of both unique and total bacterial operational taxonomic units (OTUs) were much greater in rhizospheres of OW than those in CW **(Fig 2D)**. The PCoA analysis also showed that the soil bacterial **(Fig 2E)** communities in rhizospheres of OW and CW were significant differences with the BG. Meanwhile, in the PLS-DA plots, soil bacterial compositions in rhizospheres of OW and CW, and the BG samples could be clearly distinguished and clustered into three taxa. It suggested that soil bacterial compositions in rhizospheres of OW, CW and BG were significant differences. In addition, the dispersion of the PLS-DA plots also showed that the bacterial **(Fig 2F)** composition differed less among the different treatment soils.

At the phylum level, 10 common dominant soil bacterial phyla (relative abundance value ≥ 1%) in rhizospheres of OW, CW and BG were detected as Actinobacteriota (28.74%-31.32%), Proteobacteria (23.16%-33.13%), Chloroflexi (12.42%- 16.49%), Acidobacteriota (7.14%-13.58%), Gemmatimonadota (3.78%-5.76%), Bacteroidota (1.45%-4.09%), Firmicutes (1.91%-3.80%), Myxococcota (1.42%-1.70%), Patescibacteria (less than 1%-1.89%), WPS-2 (less than 1%-1.81%) and others (2.26%-3.98%) **(Fig 2G)**. Meanwhile, Patescibacteria was the unique dominant soil bacterial phylum in rhizospheres of OW and CW, WPS-2 only was the unique dominant soil bacterial phylum in the background (BG). At the genus level, there were 19, 23, and 25 dominant rhizospheric bacterial genera in OW, CW, and BG, respectively **(Fig 2H)**. In comparison with the OW, *norank_f__SC-I-84*, *Sinomonas*, *unclassified_f__Xanthobacteraceae* and *Bryobacter* were the common dominant bacterial genera in rhizospheres between OW and CW. Meanwhile, *Bacillus*, *Rhodanobacter*, *Cupriavidus* and *Luteimonas* were the unique dominant bacterial genera in rhizospheres of CW. In contrast, *norank_f__Vicinamibacteraceae*, *Nocardioides*, *norank_f__A4b*, and *OLB13* were the special dominant bacterial genera in rhizospheres of OW.

A total of 88 bacterial clades exhibited significant differences by LEfSe in the rhizospheres s of OW, CW and BG (LDA > 3.5) **(Fig 2I)**. At the phylum level, Chloroflexi and WPS-2 significantly enriched; At the genus level, *norank_f__norank_o__Gaiellales*, *norank_f__norank_o__norank_c__AD3*, *norank_f__norank_o__Acidobacteriales*, *norank_f__norank_o__norank_c__norank_p__WPS-2*, *norank_f__norank_o__Elsterales*, *norank_f__norank_o__norank_c__TK10*, *unclassified_f__Xanthobacteraceae*, *Acidothermus*, *Conexibacter*, *norank_f__norank_o__IMCC26256*, and *Massilia* significantly enriched in background soils (BG).

At the phylum level, Bacteroidota significantly enriched; Also, at the genus level, *norank_f__JG30-KF-CM45*, *OLB13*, *Nocardioides*, *norank_f__AKYG1722*, *Ensifer*, *Saccharomonospora*, *norank_f__A4b*, and *norank_f__Vicinamibacteraceae* significantly enriched in rhizospheres of OW; In contrast, at the phylum level, Proteobacteria and Gemmatimonadota significantly enriched;, at the genus level, *Luteimonas*, *Devosia*, *Cupriavidus*, *norank_f__Gemmatimonadaceae*, *norank_f__SC-I-84*, *Rhodanobacter* significantly enriched in rhizospheres of CW.

### Soil fungal compositions in rhizospheres of watermelons with different fruit shapes

**Fig 3:**
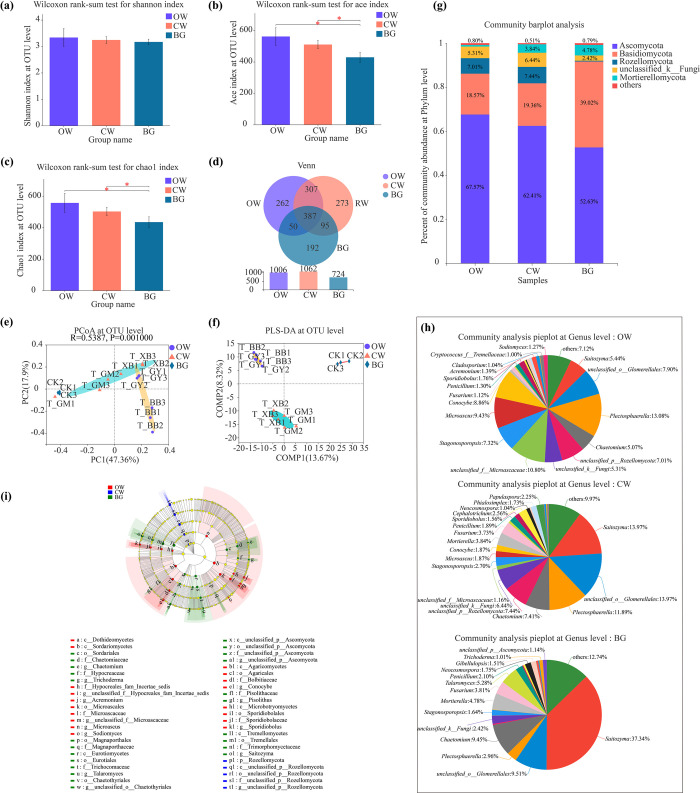
Comparison of soil fungal community structures in rhizospheres of OW, CW and BG. **(a)** The Shannon index of soil fungal diversity at the OTU level. **(b)** The Ace index of soil fungal richness at the OTU level. **(c)** The Chao 1 index of soil fungal richness at the OTU level. **(d)** PCoA of soil fungal communities at the OTU level. **(e)** PLS-DA of soil fungal communities at the OTU level. **(f)** Venn analyses of soil fungal communities at the OTU level. **(g)** Compositions of dominant soil fungi at the phylum level. **(h)** Compositions of dominant soil fungi at the genus level. **(i)** LEfSe analysis of dominant soil fungi (LDA > 3.5).

The diversity of soil fungi in rhizospheres of watermelons with different fruit shapes showed that the same trend as those of soil bacteria in rhizospheres **(Fig 3A)**. The soil fungal richness indices (Ace and Chao 1) in rhizospheres of OW and CW were also significantly higher than those of BG, although there was no statistically significant difference between OW and CW (*P* > 0.05) **(Fig 3B and 3C)**. In addition, the results of Venn analysis also showed that the numbers of unique and total soil fungal OTUs in rhizospheres of OW were all lower than those of CW **(Fig 3D)**. The PCoA analysis also showed that the soil fungal communities in rhizospheres of OW and CW were significant differences with BG **(Fig 3E)**. However, soil fungal communities in rhizospheres between OW and CW were partially overlapped. Meanwhile, in the PLS-DA plots, soil fungal compositions in rhizospheres of OW and CW, and the BG samples could be clearly distinguished and clustered into three taxa. It suggested that soil fungal compositions in rhizospheres of OW, CW and BG were significant differences. In addition, the dispersion of the PLS-DA plots also showed that the fungal **(Fig 3F)** composition differed less among the different treatments.

At the phylum level, four dominant soil fungal phyla were dominated by Ascomycota (52.63%-67.57%), Basidiomycota (18.57%-39.02%), Rozellomycota (less than 1%-7.44%), unclassified_k__Fungi (2.42%-6.44%) and Mortierellomycota (1%-4.78%) **(Fig 3G)**. Among them, Rozellomycota was the unique dominant soil fungal phylum in BG. At the genus level, there were 17, 18, and 14 dominant soil fungal genera in rhizospheres of the OW, CW, and BG, respectively **(Fig 3H)**. In comparison with OW, *Mortierella*,*Cephalotrichum*, *Neocosmospora*, *Phialosimplex*, and *Papulaspora* were the unique dominant soil fungal genera in rhizospheres of CW. *Acremonium*, *Cladosporium*, *Cryptococcus_f__Tremellaceae*, and *Sodiomyces* were the special dominant soil fungal genera in rhizospheres of OW.

Moreover, a total of 46 soil fungal clades exhibited significant differences by LEfSe in rhizospheres of OW, CW and BG (LDA > 3.5) **(Fig 3I)**. Although no significant enrichment of soil-dominant fungi could be detected at the phylum level, however, at the genus level, *Saitozyma*, *Talaromyces*, *Chaetomium*, *Pisolithus*, *unclassified_p__Ascomycota*, *Trichoderma*, and *unclassified_o__Chaetothyriales* significantly enriched in BG. Meanwhile, at the phylum level, also, no significant enrichment of dominant soil fungi could be detected, however, at the genus level *unclassified_f__Microascaceae*, *Microascus*, *Conocybe*, *Sporidiobolus*, *Acremonium*, *Sodiomyces*, and *unclassified_f__Hypocreales_fam_Incertae_sedis* significantly enriched in rhizospheres of OW. Also, at the phylum level, Rozellomycota, and at the genus level, *unclassified_p__Rozellomycota* significantly enriched in rhizospheres of CW.

### Endophytic bacterial compositions in stems of watermelons with different fruit shapes

**Fig 4:**
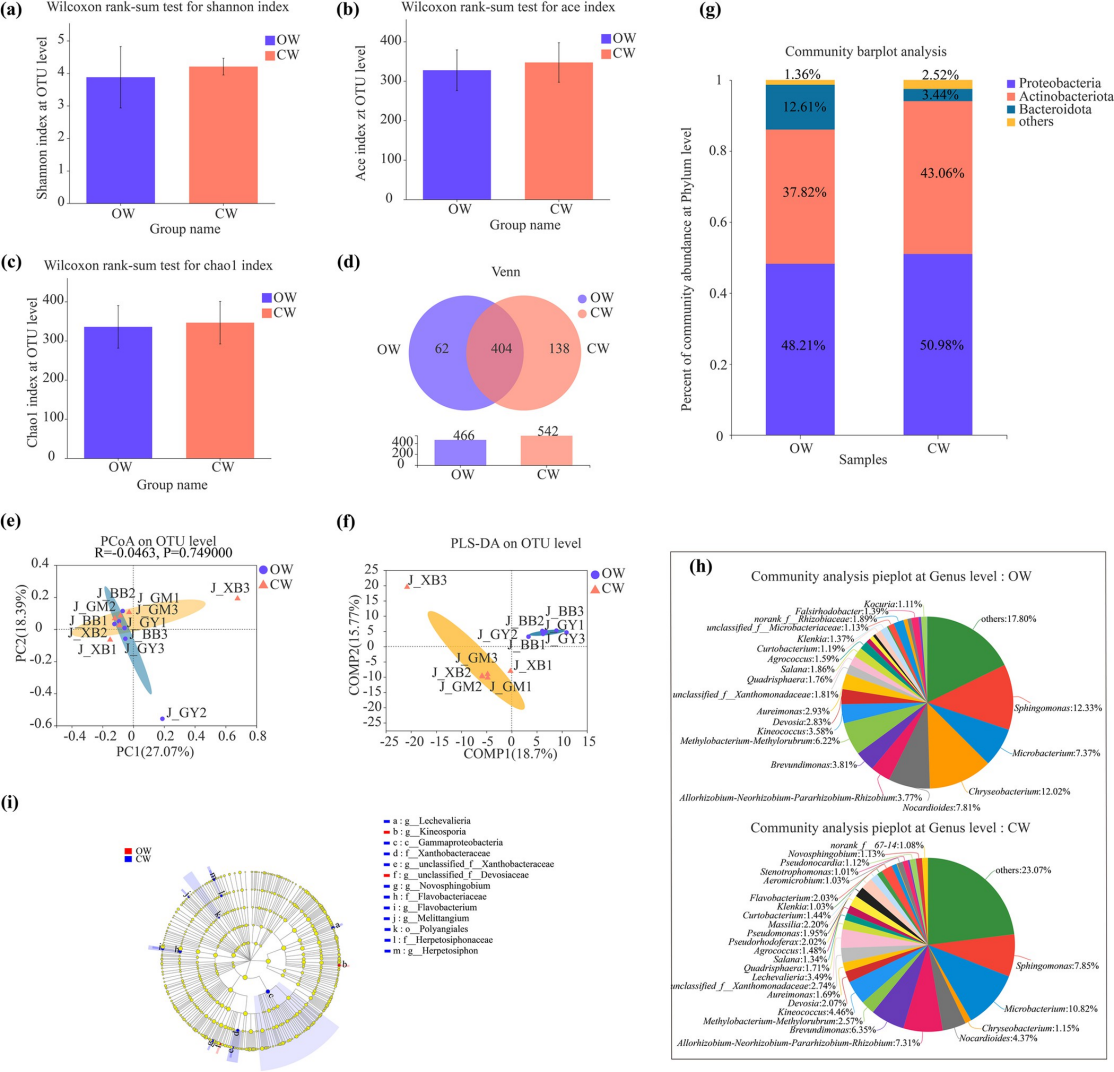
Comparison of endophytic bacterial community structures in stems between oval (OW) and circular watermelons (CW). **(a)** The Shannon index of endophytic bacterial diversity at the OTU level. **(b)** The Ace index of endophytic bacterial richness at the OTU level. **(c)** The Chao 1 index of endophytic bacterial richness at the OTU level. **(d)** PCoA of endophytic bacterial communities at the OTU level. **(e)** PLS-DA of endophytic bacterial communities at the OTU level. **(f)** Venn analyses of endophytic bacteria for the two treatments at the OTU level. **(g)** Compositions of dominant endophytic bacterial communities at the phylum level. **(h)** Compositions of dominant endophytic bacterial communities at the genus level. **(i)** LEfSe analysis of dominant endophytic bacteria (LDA > 2.0).

The endophytic bacterial diversity (Shannon) and richness (Ace and Chao 1) indexes were all not statistically significant between OW and CW (*P* > 0.05) **(Fig 4A–4C).** In addition, the numbers of both unique and total endophytic bacterial OTUs were much less in stems of OW than those of CW **(Fig 4D)**. Base on the PCoA and the PLS-DA analyses, the endophytic bacterial communities were mostly partially overlapped and were not significant differences between OW and CW (*P* > 0.05) **(Fig 4E and 4F)**. Meanwhile, Proteobacteria (48.21%), Actinobacteriota (37.82%), Bacteroidota (3.44%) and others (1.36%) were detected as the dominant endophytic bacterial phyla in stems of oval watermelon (OW); By contrast, Proteobacteria (50.98%), Actinobacteriota (43.06%), Bacteroidota (12.61%) and others (2.52%) were also found as the dominant endophytic bacterial phyla in stems of circular watermelon (CW) **(Fig 4G)**. Additionally, there were 20 and 26 dominant endophytic bacterial genera in stems of OW and CW, respectively **(Fig 4H)**. *Lechevalieria*, *Pseudorhodoferax*, *Pseudomonas*, *Massili*a, *Flavobacterium*, *Aeromicrobium*, *Stenotrophomonas*, *Pseudonocardia*, *Novosphingobium*, and *norank_f__67–14* were the unique dominant endophytic bacterial genera in stems of CW. In contrast, *unclassified_f__Microbacteriaceae*, *norank_f__Rhizobiaceae*, *Falsirhodobacter*, and *Kocuria* were the special dominant endophytic bacterial genera in stems of OW.

Meanwhile, at the phylum level, there were no any significant enrichments of endophytic bacteria in both OW and CW (LDA > 2.0) **(Fig 4I)**. However, at the genus level, *Kineosporia* and *unclassified_f__Devosiaceae* significantly enriched in stems of OW; In contrast, *Lechevalieria*, *unclassified_f__Xanthobacteraceae*, *Novosphingobium*, *Flavobacterium*, *Melittangium*, *Herpetosiphon* significantly enriched in stems of CW.

### Endophytic fungal compositions in stems of watermelons with different fruit shapes

**Fig 5:**
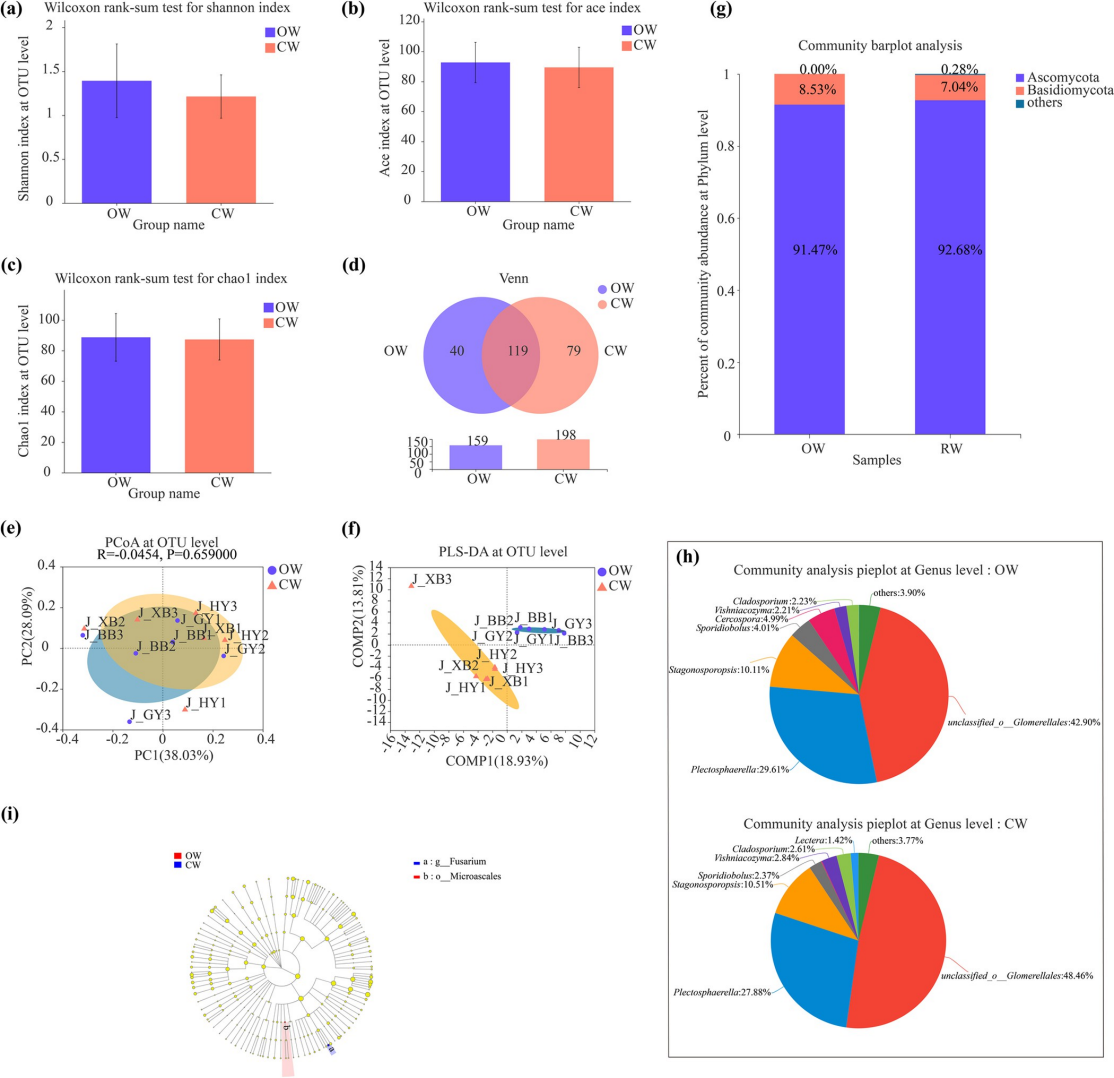
Comparison of endophytic fungal community structures in stems between oval (OW) and circular watermelons (CW). **(a)** The Shannon index of endophytic fungal diversity at the OTU level. **(b)** The Ace index of endophytic fungal richness at the OTU level. **(c)** The Chao 1 index of endophytic fungal richness at the OTU level. **(d)** PCoA of endophytic fungal communities at the OTU level. **(e)** PLS-DA of endophytic fungal communities at the OTU level. **(f)** Venn analyses of endophytic fungi at the OTU level. **(g)** Compositions of dominant endophytic fungi at the phylum level. **(h)** Compositions of dominant endophytic fungi at the genus level. **(d)** LEfSe analysis of dominant endophytic fungi (LDA > 2.0).

As shown at the **Fig 5A–5C,** the endophytic fungal diversity (Shannon) and richness (Ace and Chao 1) indexes were all not significantly different in stems between OW and CW (*P* > 0.05). Meanwhile, the numbers of unique and total endophytic fungal OTUs were much less in stems of OW than those of in CW **(Fig 5D)**. Base on the PCoA and the PLS-DA analyses, the endophytic fungal communities were mostly partially overlapped and not significant differences between OW and CW (*P* > 0.05) **(Fig 5E and 5F)**.

Additionally, the dominant endophytic fungal phyla in stems of OW were Ascomycota (91.47%) and Basidiomycota (8.53%); By contrast, Ascomycota (92.68%) and Basidiomycota (7.04%) were also the dominant endophytic fungal phyla in stems of CW **(Fig 5G)**. i.e., only the proportions of dominant endophytic phyla were different in stems of OW and CW. Meanwhile, there were all 7 dominant endophytic fungal genera in stems of OW and CW. However, *Lectera* was the unique dominant endophytic fungal genus in stems of CW; In contrast, *Cercospora* was the unique dominant endophytic fungal genus in stems of OW **(Fig 5H)**.

Moreover, at the phylum level, not any endophytic fungal phylum significantly enriched in stems of OW and CW (LDA > 2.0). Furthermore, at the genus level, not any endophytic fungal genus significantly enriched in stems of OW; By contrast, *Fusarium* significantly enriched in stems of CW **(Fig 5I)**.

### Phenotypic predictions of the rhizospheric and endophytic bacteria

**Fig 6:**
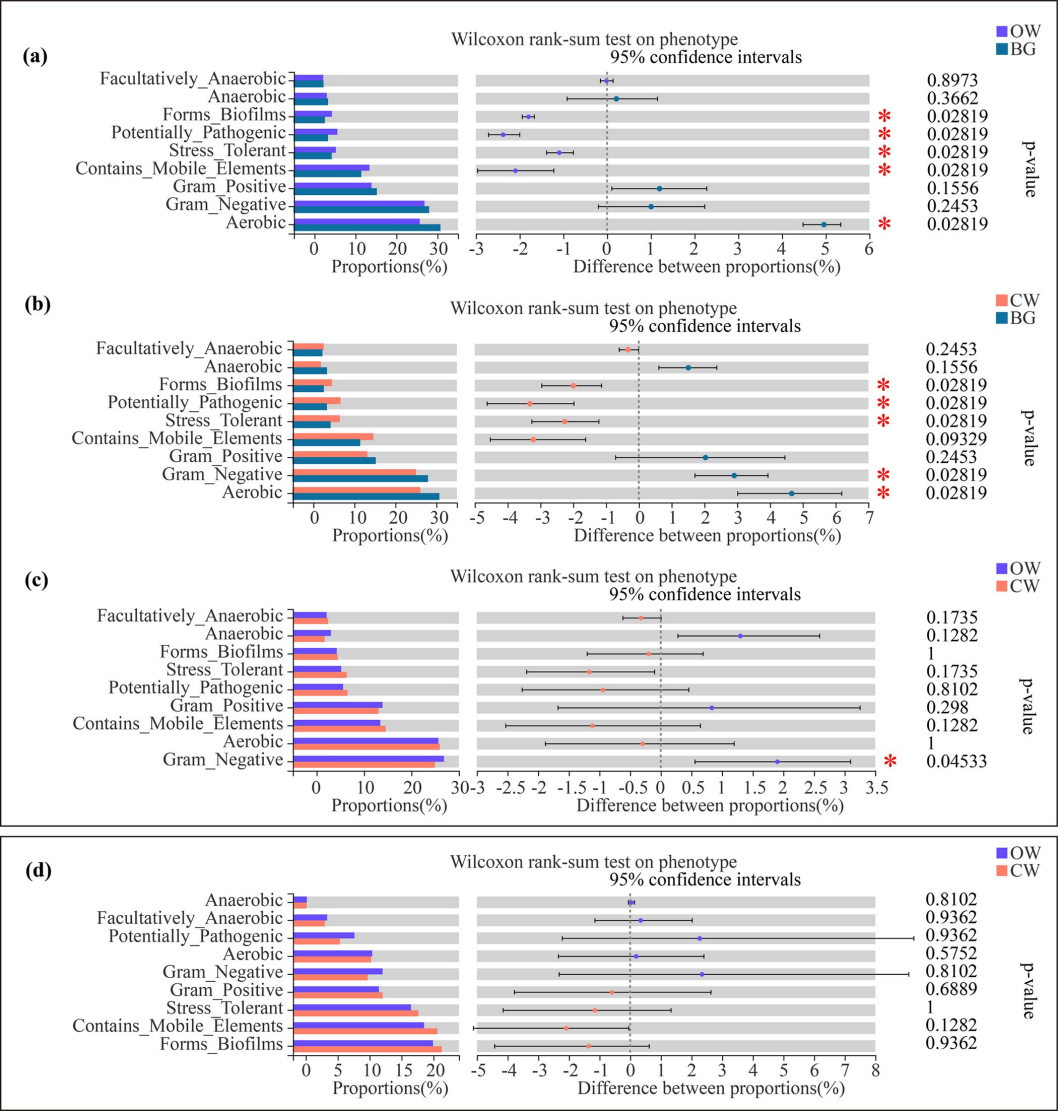
Phenotypic predictions of dominant rhizospheric (a, b, c) and endophytic (d) bacteria. *0.01 < *P* ≤ 0.05 (Wilcoxon rank-sum test).

The results showed that the Forms Biofilms, Potentially Pathogenic, Stress Tolerant, Contains Mobile Elements and Aerobic of soil bacteria in rhizospheres between OW and CW were all significantly different with those of BG **(Fig 6A and 6B)**. Additionally, Gram Negative soil bacteria in rhizospheres of OW were also significantly higher than those of CW **(Fig 6C)**. However, there were not significant difference of nine endophytic bacterial functions in stems between OW and CW (*P* > 0.05) **(Fig 6D)**.

## Discussion

Previous studies had confirmed that fruit shape was depended on cell division and enlargement [5,22,23]. During the growth and development period of pomegranate, the fruit shape gradually changed from elongated oval in the early stage to circular in the maturity stage [24]. The process of fruit shape formation and ripening requires the involvement of various hormones such as auxin, cytokinins, gibberellins, abscisic acid and ethylene [25,26]. For example, gibberellic acids (GAs) were well correlated with cell division and expansion [26,27]. Eriksson et al. [28] found that higher endogenous GA levels promoted more and longer cells, fruit elongation could be induced by higher exogenous GA3s levels [26]. Southwick and Glozer [29] also found that gibberellins could increase fruit size, such as fruits shapes of grapes could be affected by GA. Christodoulou et al. [30] found that the treatment of young grapes with GA caused the fruit growing longitudinally which resulted in a long oval shape. Meanwhile, Liao et al. [31] found that fruits widths and lengths growing all could be stimulating by auxin, but gibberellin [gibberellic acid (GA)] mainly promotes their longitudinal elongation. However, GA biosynthesis and signaling genes could be activated by auxin. For example, cucumbers, GA could antagonize IAA to inhibit fruit elongation in the mid-to-early stage. i.e., plant hormones could work together in regulating fruit development and shape [32].

As the rhizospheric soil dominant bacteria of yellow and green rinds watermelons were mainly composed with Actinobacteriota, Proteobacteria, Chloroflexi, Acidobacteriota, Gemmatimonadota [33]. And the endophytic dominant bacteria in stems of yellow and green rinds watermelons were composed of Proteobacteria, Actinobacteriota and Bacteroidota [33]. Our results also revealed that Actinobacteriota, Proteobacteria, Chloroflexi, Acidobacteriota, Gemmatimonadota were the five dominant soil bacterial phyla in rhizosphere, and Proteobacteria, Actinobacteriota, Bacteroidota were also the three dominant endophytic bacterial phyla in stems of OW and CW, respectively. As the most active auxin in plants, indole acetic acid (IAA) could be produced by *Bacillus* [34,35]. We found that *Bacillus* was the unique soil dominant bacteria in rhizospheres of CW. Moreover, as IAA and gibberellins could be produced by *Fusarium proliferatum* BRL1 [36]; And IAA also could be generated by *Fusarium tricinctum* RSF-4L, *Penicillium chrysogenum* and *Penicillium crustosum* [37,38]. Furthermore, gibberellin (GA) and IAA also could be produced by *Penicillium* sp. LWL3 [39]. *Mortierella* could product and accumulate IAA in its mycelia, meanwhile, it also could product the organic acids to desorb phosphorus in contributing to bioavailable phosphorus in the environment [40]. *Mortierella* was the unique dominant rhizospheric fungi in the CW. In our study, we found that the proportions of *Fusarium*, *Penicillium*, and *Mortierella* in rhizospheres of CW were all higher than those of OW. Therefore, higher contents of IAA and GA could be speculated in rhizospheres of CW.

In addition, the longitudinal elongations of tomato and woodland strawberry could be inhibited by endogenous abscisic acid (ABA) [31,41]. However, ABA content was significantly and positively correlated with the percentage of Actinobacteriota [42]. We found that higher proportions of Actinobacteriota could be detected in stems of CW than those of OW. In addition, Bučková et al. [43] found that *Cladosporium cladosporioides* could produce ABA. Our results showed that the percentage of *Cladosporium* in stems of CW was higher than that of OW. Therefore, it could be speculated that higher ABA content in stems of circular watermelons (CW) might inhibit longitudinal growth of their fruits.

It is inferred that the different rhizospheric or endophytic microorganisms enriched in rhizospheres and stems between oval and circular watermelon varieties may further induce different gene functions by changing the external (hormones, enzymes, nutrient levels, etc.) and internal (endogenous hormone types and contents, enzymes, internal nutrient levels, etc.) environments of watermelon with different fruit shapes. As a result, various soil and endophytic microorganisms enriched in rhizospheres or stems of watermelons with different fruit shaped forming their special micro-environments for watermelons growth, respectively. In other words, soil and endophytic microorganisms in rhizospheres or stems of watermelons can be speculated in relating to watermelon fruit shapes formation.

## Conclusions

*Bacillus*, *Rhodanobacter*, *Cupriavidus*, *Luteimonas*, and *Devosia* were the unique soil dominant bacterial genera in rhizospheres of circular watermelon (CW); In contrast, *Nocardioides*, *Ensifer*, and *Saccharomonospora* were the unique soil dominant bacterial genera in rhizospheres of oval watermelons (OW); Meanwhile, *Cephalotrichum*, *Neocosmospora*, *Phialosimplex*, and *Papulaspora* were the unique soil dominant fungal genera in rhizospheres of circular watermelon (CW); By contrast, *Acremonium*, *Cladosporium*, *Cryptococcus_f__Tremellaceae*, *Sodiomyces*, *Microascus*, *Conocybe*, *Sporidiobolus*, and *Acremonium* were the unique soil dominant fungal genera in rhizospheres of oval watermelons (OW). Additionally, *Lechevalieria*, *Pseudorhodoferax*, *Pseudomonas*, *Massili*a, *Flavobacterium*, *Aeromicrobium*, *Stenotrophomonas*, *Pseudonocardia*, *Novosphingobium*, *Melittangium*, and *Herpetosiphon* were the unique dominant endophytic bacterial genera in stems of CW; In contrast, *Falsirhodobacter*, *Kocuria*, and *Kineosporia* were the special dominant endophytic genera in stems of OW; Moreover, *Lectera* and *Fusarium* were the unique dominant endophytic fungal genera in stems of CW; By contrast, *Cercospora* only was the special dominant endophytic fungal genus in stems of OW. All above results suggested that watermelons with different fruit shapes exactly recruited various microorganisms in rhizospheres and stems. Meanwhile, the enrichments of the different rhizosphric and endophytic microorganisms could be speculated in relating to watermelon fruit shapes formation.
